# Diabetes Resolution at 10 Years After Biliopancreatic Diversion in Overweight and Class 1 Obese Patients with Type 2 Diabetes

**DOI:** 10.1007/s11695-021-05870-4

**Published:** 2022-01-10

**Authors:** Francesco Saverio Papadia, Flavia Carlini, Alice Rubartelli, Micaela Battistini, Renzo Cordera, Gian Franco Adami, Giovanni Camerini

**Affiliations:** 1 Department of Surgery, University of Genova, Genova, Italy; 2 Department of Internal Medicine, University of Genova, 8, Viale Benedetto XV, 16132 Genova, Italy

**Keywords:** Type 2 diabetes, Non-morbid obesity, Metabolic surgery, Biliopancreatic diversion, Long-term results

## Abstract

**Background:**

Long-term anti-diabetic effects of BPD in overweight or class 1 obese T2DM patients were investigated reporting the results at 10 years after BPD performed in severely non-obese T2DM patients.

**Material and Methods:**

Thirty T2DM patients with BMI lower than 35 kg/m^2^ were investigated at 1, 5, and 10 years after BPD, and the results are compared with those of 30 T2DM patients followed for 10 years on pharmacological and/or behavioral conventional therapy.

**Results:**

Mean levels of fasting blood glucose (FBG) and serum glycated hemoglobin (HbA1_C_) showed a marked reduction 1 year after BPD, values remaining slightly above the diabetic range throughout the entire follow-up. T2DM remission was observed in about 50% of the cases at 5 and 10 years after the operation. In 16 patients (53%), severe BPD-related complications developed, in ten cases requiring a surgical revision of the operation. In the BPD group, one patient died for malignant lymphoma and two patients after surgical revision. Within the control group, during the 10-year follow-up, no changes in the diabetic status were observed, being the FBG and HbA1_C_ mean values higher than those recorded in the BPD patients at any follow-up time. All T2DM subjects of the control group were alive at the end of the 10-year follow-up.

**Conclusion:**

Despite satisfactory long-term metabolic outcomes, these data indicate that BPD should be used with caution as a metabolic procedure in the treatment of T2DM in overweight or class 1obese patients.

**Graphical abstract:**

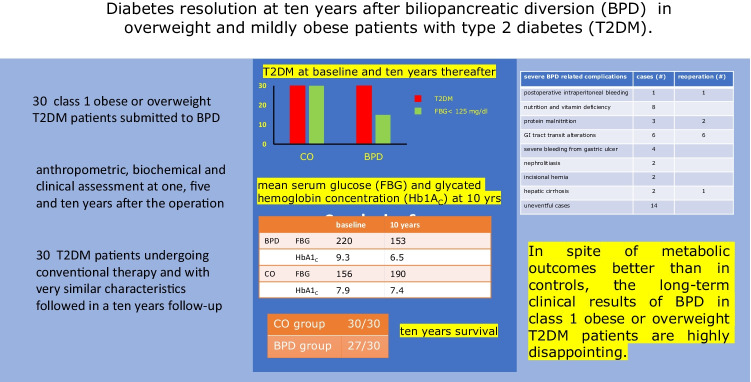

In type 2 diabetic (T2DM) severely obese patients, recent randomized and controlled trials have confirmed that, in achieving and maintaining glycemic control, metabolic/bariatric surgery is far more efficient than the traditional pharmacological conservative therapies [1–5]. Furthermore, beneficial effects of metabolic/bariatric surgery are not limited to blood glucose control: after the operation, dyslipidemia tends to disappear, arterial hypertension subsides towards normality, the occurrence of cardiovascular diseases decreases, and the overall mortality is reduced [3, 5–12]. Functional studies demonstrated that the metabolic benefits become evident early after the operation when body weight is still in the obese range [12–17]. It has thus been suggested that the satisfactory metabolic outcomes observed following bariatric surgery are due to the specific effects of the operations, more than to the weight loss and the resulting reduction of insulin resistance. Therefore, in recent years, several studies have been carried out to investigate the possible role of metabolic/bariatric surgery in the treatment of T2DM in class 1 obese or simply overweight subjects. The available findings demonstrate that in class 1 obese and overweight patients, bariatric/metabolic surgery is followed by a T2DM remission or by a noticeable improvement of glucose control in a significant number of operated subjects, the results being overall better than those observed in T2DM individuals treated by medical/behavioral therapy [17–21].

As far as a biliopancreatic diversion (BPD) is concerned, the greater is the baseline degree of obesity, the higher is the rate of patients showing a postoperative stable T2DM remission or an improvement of glucose control [22]. In T2DM class 1 or overweight subjects, the metabolic outcomes obtained following BPD were overall similar to those reported after other bariatric procedures [23].

This paper has three main aims: (a) evaluating the stability of the metabolic outcome observed 5 years after BPD, recording the clinical results of the original cohort of T2DM overweight and class 1 obese patients submitted to BPD at 10 years following the operation; (b) considering the occurrence and the clinical course of the long-term complications related both to T2DM and to BPD by itself throughout the entire follow-up period; (c) investigating the rate of T2DM complications and the overall mortality in the BPD individuals by comparing findings recorded throughout the postoperative follow-up with the clinical ones observed in a group of T2DM patients conventionally treated during the same period.

## Material and Methods

The investigation was carried out in the original 30 patients submitted to bariatric surgery for T2DM treatment from September 2007 to February 2008, who were specifically operated on to assess the clinical outcome and the functional changes on glucose metabolism after BPD in class 1 obese and overweight T2DM [24]. The inclusion criteria were the following: the presence of overt T2DM for more than 3 years, glycosylated hemoglobin (Hb1A_C_) higher than 7.5% despite specific anti-diabetic therapy, basal body mass index (BMI) comprised between 25 and 35 kg/m^2^, age from 35 to 70 years, no pregnancy and malignancy, and absence of clinical or social conditions that could prevent a regular postoperative follow-up. Positivity for anti-islet antibodies, C-peptide lower than 0.5 ng/ml and clinical or biochemical suspect of latent autoimmune diabetes in adults (LADA) was a strong exclusion criterion. All patients signed their informed consent to the operation and to the anonymous data processing. The study protocol was approved by the local Ethical Committee and the study was registered (NCT00996294).

A group of thirty T2DM subjects undergoing behavioral, dietetic, and pharmacologic therapy for at least 10 years at the San Martino Hospital Diabetes Service (CO group), matched to those of the surgical group (BPD group) for age, gender, BMI, and with similar T2DM duration, served as controls.

All patients were submitted to the standard type of BPD, with the alimentary limb of 250 cm and the common limb of 50 cm. Peri- and postoperative treatment was the standard one for BPD at our institution [25].

For this investigation, only the findings recorded before the operation and at 1, 5, and 10 years following BPD were considered. The follow-up rate at 10 years was 100%.

Body weight (BW) was determined to the nearest 0.1 kg and stature to the nearest 0.5 cm. Fasting blood glucose (FBG, mg/dl) and glycated hemoglobin (Hb1A_C_, %) concentrations were measured with a routine analyzer. Standard methods were employed for determining serum levels of triglycerides, total cholesterol, and LDL cholesterol.

Arterial hypertension was defined as a systolic blood pressure of more than 140 mm Hg and/or diastolic blood pressure of more than 85 mm Hg or chronically taking antihypertensive medication.

When serum creatinine was higher than 2 mg/dl, the patients were considered as having chronic kidney insufficiency (CKI).

Following the operation, T2DM was regarded as remitted when serum FBG concentration was steadily lower than 125 mg/ml at free diet and without taking any anti-diabetic medication [26].

In the operated patients, the anthropometric, biochemical, and clinical data were collected at 1, 5, and 10 years after BPD.

The development of BPD-related early or late complications (severe intraperitoneal postoperative bleeding, nutrition and vitamin deficiency, protein malnutrition, gastrointestinal transit alterations and chronic diarrhea, bleeding from gastric ulcer, nephrolithiasis, incisional hernia, and hepatic cirrhosis), that in some cases have required revisional surgery, was recorded.

At the tenth year after the operation, the patients were specifically asked for the presence of diabetes complications (retinopathy, neuropathy, KCI, and diabetic foot) and the history of cardiovascular disease, as acute myocardial infarction (IMA), stroke, and chronic lower limb ischemia (CLLI).

The control subjects underwent an anthropometric, biochemical, and clinical assessment at the basal time and after the 10-year follow-up period, when the prevalence of T2DM complications was recorded.

Cross-sectional differences were assessed with the Mann–Whitney U test for independent comparisons, while longitudinal changes were evaluated with the Wilcoxon rank test; categorical data were analyzed with the classical and with the Mac Nemar chi-square test, when appropriate.

## Results

In Table 1, the results obtained in the whole cohort of the operated subjects are referred to. A marked decrease in BW and BMI mean values was observed at 1 year after the operation, and the values remained essentially stable at 5 and 10 years after BPD, without any appreciable change in percentage of weight loss (%EWL) throughout all the follow-up. Likewise, a marked reduction of FBG and Hb1A_C_ serum concentration was observed 1 year after the operation, with mean values remaining essentially unchanged at all the follow-up points. However, the mean FBG and Hb1A_C_ values were still slightly above the diabetic level at 1, 5, and 10 years following BPD. At the 1-year control, nine patients (30%) have normalized FBG at free diet and without specific therapy, while subsequently the number of patients showing a normal FBG level increased at 5 and 10 years (59 and 48%, respectively), i.e., nearly half of the operated patients showed a complete T2DM remission at the end of the 10-year follow-up (Table 1). While preoperatively all patients were on anti-diabetic medication, throughout the follow-up period, only half of the patients were currently on oral agents, while the rate of insulin use remained nearly unchanged, although at markedly reduced doses.

**Table 1:** Type two diabetes (T2DM) class 1 obese and overweight patients: anthropometric, biochemical, and clinical data prior to, at 5 and at 10 years following biliopancreatic diversion. *BMI*, body mass index; *BW*, body weight; *%EWL*: percentage excess weight lost; *FBG*, fasting blood glucose; Hba1_C_, glycosylated hemoglobin. T2DM was considered as remitted when FBG is steadily lower than 125 mg/dl without antidiabetic agents

	Prior to BPD	At 1 year after BPD	At 5 years after BPD	At 10 years after BPD
Cases (#)	30	30	29	27
Age (years)	56.7 (35–70)			
Gender (male)	19 M			
T2DM duration (years and range)	11.1 (3–15)			
Antidiabetic therapy (# and %)	30 (100)	21 (80)*	16 (55)*	12 (40)*
Insulin use (# and %)	15 (50)	16 (53)	13 (45)	12 (40)
BMI (kg/m^2^, mean ± sd)	30.6 ± 2.9	25.3 ± 2.3*	25.7 ± 2.8*	24.9 ± 2.4*
BW (kg, mean ± sd)	84.8 ± 11.1	70.7 ± 10.2*	73 ± 10.2*	68.5 ± 9.2*
EWL (%, mean ± sd)		24.8 ± 11.9	23.3 ± 12	25.6 ± 14.2
FBG (mg/day, mean ± sd l)	220 ± 69	149 ± 41*	140 ± 32.1*	140 ± 40.7*
HbA1_C_ (%, mean ± sd)	9.3 ± 1.5	6.3 ± 0.8*	6.6 ± 2.1*	6.5 ± 1.3*
Presence of T2DM (# and %)	30 (100)	21 (70)*	12 (41)*§	14 (52)*§
Triglycerides (mg/dl, mean ± sd	198 ± 226	204 ± 109	164 ± 81	145 ± 90
Cholesterol (mg/dl, mean ± sd)	190 ± 48	147 ± 32*	144 ± 41*	125 ± 30*
HDL cholesterol (mg/dl, mean ± sd)	47 ± 122	44 ± 10	46 ± 14	47 ± 10
Blood hypertension (# and %)	16 (53)	6 (20)*	3 (10)*	2 (7)*

^*^p < 0.01 vs. prior to BPD

^§^*p* < 0.01 vs. at 1 year after BPD

Following BPD serum triglycerides mean value was markedly reduced and normalized at 5 and 10 years. Mean serum cholesterol concentration early fell into the normal range and remained stable up to 10 years, while the serum HDL level was within physiological values both before and after the operation (Table 1).

Before the operation, 16 patients (53%) had arterial hypertension, while at 10 years following BPD in most of the subjects the blood pressure was in the normal range (22 cases, 81%).

Among the operated patients, three patients died following the operation (at three, six, and eight postoperative years, respectively), two of them for BPD-related complications.

In Table 2, the anthropometric and biochemical data of the CO subjects at the enrollment and after the 10-year follow-up period is referred to. Except for a light and not significant weight gain, all baseline data were essentially unchanged 10 years later. Both at the starting point and the end of the follow-up, all patients currently assumed antidiabetic therapy and half of them received insulin. No death was observed among the control subjects.

**Table 2:** Type 2 diabetes (T2DM) patients undergoing behavioral, dietetic, and pharmacological treatment (CO subjects). Anthropometric, biochemical, and clinical data at the enrollment and after a 10-year follow-up. *BMI*, body mass index; *BW*, body weight; *FBG*, fasting blood glucose; *Hba1*_*C*_, glycosylated hemoglobin. T2DM was considered as remitted when FBG is steadily lower than 125 mg/dl without antidiabetic agents

	Basal evaluation	At 10-year follow-up
Age (years)	59 ± 4.5	
Gender (male)	22	
T2DM duration (years and range)	8 (4–12)	
Presence of T2DM	30	30
Antidiabetic therapy (#)	30	30
Insulin use (# and %)	13 (43)	15 (50)
BMI (kg/m^2^, mean ± sd)	28.9 ± 3.28	31.6 ± 2.58
BW (kg, mean ± sd)	82.6 ± 10.1	84.8 ± 14.6
FBG (mg/day, mean ± sd l)	156 ± 45	190 ± 42
HbA1_C_ (%, mean ± sd)	7.9 ± 0.37	7.43 ± 0.85
Triglycerides (mg/dl, mean ± sd	141 ± 74	102 ± 58
Cholesterol (mg/dl, mean ± sd)	189 ± 30	182 ± 34.6
HDL cholesterol (mg/dl, mean ± sd)	42 ± 8.3	43 ± 11

At the baseline, all clinical and biochemical data were quite similar in the CO and the BPD individual, while at the end of the follow-up period, in the BPD subjects significantly lower mean values of FBG and HbA1_C_ (*p* < 0.05 and *p* < 0.01, respectively) were observed; furthermore, in comparison with the BPD patients, in control subjects, lesser antidiabetic agents use (*p* < 0.01) was recorded, with no difference in insulin treatment (Tables 1 and 2).

At the end of the follow-up period, the prevalence of T2DM complications reported by the subjects submitted to metabolic surgery (BPD group) was nearly similar to that described in control subjects (CO group), and likewise in the two groups, the number of the no-complications individuals was quite similar (Table 3). No death was observed in the CO group.

**Table 3:** Type 2 diabetes (T2DM) complications in class 1obese and overweight T2DM patients undergoing biliopancreatic diversion (BPD group) and in a control group of T2DM patients undergoing medical and behavioral therapy for 10 years (CO group)

	CO group	BPD group
T2DM complications		
Retinopathy	6	5
Neuropathy	1	2
Chronic kidney insufficiency	2	4
Diabetic foot		1
No complication (subjects)	12	16

Throughout the follow-up period, the mortality rate in the patients having undergone BPD was significantly higher than in the control individuals (*p* < 0.001).

Among the entire group of the operated subjects, the postoperative follow-up was substantially uneventful in 14 cases, while in the other subjects one or more BPD-related severe complications were observed, in ten cases requiring re-operation (Table 4). One patient had intraperitoneal bleeding in the postoperative period and underwent explorative laparotomy the day after the operation. In eight cases, a vitamin or oligo-elements deficiency developed. In three cases, true protein malnutrition occurred, requiring specific parenteral support, and in two patients, a BPD revision was indicated. Severe diarrhea was reported by six patients, in two cases necessitating BPD revision and in four cases a complete BPD restoration. Acute gastrointestinal bleeding from peptic ulcer needing a blood transfusion and intensive care occurred in four subjects. In one case, BPD was followed by nephrolithiasis and in two cases by incisional hernia. One patient developed postoperative alcohol-related hepatic cirrhosis that needed a complete BPD restoration. One patient developed lung cancer 8 years after BPD, and one patient had acute leukemia, which caused its death 6 years after the operation. Two patients died due to postoperative complications after revisional surgery. The occurrence of BPD-related complications was similar in the subjects demonstrating long-term T2DM remission (six complicated and six uneventful cases) and in the metabolically unsuccessful ones (ten complicated and eight uneventful cases).

**Table 4:** Type two diabetes (T2DM) class 1 obese and overweight patients having undergone biliopancreatic diversion (BPD): occurrence of BPD-related complication throughout the 10-year follow-up period

BPD-related complication	Cases (#)	Reoperation (# of subjects)		
		Laparotomy	Elongation	Restoration
Postoperative intraperitoneal bleeding	1	1		
Nutrition and vitamin deficiency	8			
Protein malnutrition	3		2	
Gastrointestinal transit alterations	6		2	4
Severe bleeding from gastric ulcer	4			
Nephrolithiasis	1			
Incisional hernia	2			
Hepatic cirrhosis	1			1
Uneventful (# and % of subjects)	14 (46)			

## Discussion

The first result of this study is that the postoperative biochemical results obtained in the short-term following BPD are substantially maintained for the very long term following the operation. In comparison with the 1-year data [24], the T2DM remission rate at 5 years following BPD increases and tends to remain unchanged at a very long term: at 10 years following the operation, nearly half of operated patients still have a normal glucose level without any antidiabetic therapy. Furthermore, the serum FBG and Hb1Ac mean values observed at 1 year after BPD are substantially maintained throughout all the follow-up periods, without any tendency to the biochemical T2DM relapse. Moreover, in the BPD subjects, the data are far better in terms of long-term T2DM remission rate, use of antidiabetic medication, FBG, and HbA1c mean concentration than those observed in a control group fully comparable to the BPD one for age, gender, T2DM duration, insulin use, and BMI level. The results of this study are in keeping with those of previous controlled investigations carried out in a similar population with different surgical procedures [20, 27–31]. Therefore, BPD seems to lead to a stable T2DM remission in most T2DM overweight and class 1 obese subjects.

In terms of T2DM remission, the metabolic outcomes of BPD in T2DM severely obese patients are far better than those observed in this study carried out on overweight and class 1 obese patients; in the morbidly obese patients, the long-term diabetes remission after BPD being the rule [4, 16, 32–35]. In severely obese individuals, beta-cell mass is expanded [36, 37], and yet in the early postoperative period, a marked increase of insulin secretion develops, thus strongly contributing to the recovery of a normal metabolic status [14, 15]. On the contrary, it can be hypothesized that in most T2DM severely non-obese patients, due to a defective beta-cell system, the deficit of insulin secretion cannot be reverted after BPD, so that a positive metabolic outcome would be substantially accounted for entirely by the postoperative reduction of insulin resistance [22, 23, 38].

In the severely obese patients having undergone metabolic/bariatric surgery, a marked decrease of micro and macrovascular T2DM complications was observed [6–9, 12], while in this study, the occurrence of T2DM-related complications during the 10-year follow-up period was closely similar in the BPD patients and the subjects undergoing conventional therapy. The small sample size and the consequently limited number of complications in both the BPD and CO group could make the data of this study not fully reliable. However, this could raise the hypothesis that in severely obese individuals, the benefits of bariatric/metabolic operations are due more to the massive weight loss than to the normalization of insulin action.

Unexpectedly, in this study, a very high number of moderate to severe complications specifically related to BPD were observed. In one case, an immediate postoperative complication requiring laparotomy occurred, while in the other cases complications developed over time throughout the follow-up. The complications hereby observed were the same as those usually observed after the standard type BPD carried out in morbidly obese patients [25] but the occurrences have been far more severe and frequent than those observed in the non-diabetic morbidly obese operated patients. Protein and symptomatic vitamin deficiency occurred in more than one-third of the cases, and in two patients with protein malnutrition, a BPD revision was required. Likewise, intestinal transit alterations with severe and intractable diarrhea were observed in six patients, and in all cases, a surgical revision was performed. Severe gastrointestinal bleeding from peptic ulcer was observed in four patients, always necessitating hospitalization, intensive care, and blood transfusions. In one patient, postoperative alcoholic liver cirrhosis was developed, needing surgical revision of BPD. In one case, nephrolithiasis with non-diabetes-related kidney chronic insufficiency was developed. Overall, only 47% of the operated patients did not experience any complications. Furthermore, the occurrence of BPD-related complications was fully unrelated to the post-BPD long-term FBG normalization.

This investigation shows that BPD has a higher complication rate in class 1 obese or overweight T2DM patients than in both diabetic and non-diabetic severely obese subjects [25, 39].

In the long and very long term, BPD is a highly demanding operation, mainly for the resulting mild and selective malnutrition of vitamins and proteins consequent to the deep rearrangement of the gastrointestinal tract. In severely obese patients, these conditions warrant satisfactory weight loss and long-term weight maintenance, necessitating however adequate behavior changes, an appropriately increased food intake, and plentiful substrate storage in the body. The average severely obese patients undergoing BPD have an average low age and so they are prone to eating behavioral changes for adapting themselves to the new gastrointestinal tract anatomo-functional conditions ensuing from BPD. Furthermore, severely obese patients are generally strong eaters, so that they can easily and unwittingly counterbalance any defective intestinal absorption of proteins or vitamins. In addition, the overrepresentation of energy and protein depots ensures against any severe temporary shortage. These conditions are lacking in class 1 obese and overweight T2DM subjects of this investigation: in fact, besides the intrinsic friability and tendency to catabolism of any T2DM subject by itself, the T2DM patients of this study had a lower degree of obesity, higher age, and most likely a lower food intake than the typical severely obese patients undergoing BPD, and this could account for the higher occurrence of severe BPD-related complications observed throughout the follow-up.

In this study, the global mortality rate of the operated patients was noticeably higher than that of the control individuals, and two of three deaths are specifically related to BPD late complication. The small sample size prevents any accurate statistical analysis, but these yet preliminary data clearly emphasize enough that in any case BPD has to be considered with great caution as a therapeutic option for T2DM in class 1 or overweight patients.

The complete and very strict clinical follow-up and the presence of a control group are the strengths of this investigation. Its great limitation is the very small sample size.

In conclusion, this study indicates that in class 1 or overweight T2DM patients submitted to BPD, the positive short-term biochemical results are maintained at 10 years after the operation and are better than those observed after conventional therapy. However, the clinical results are highly disappointing, and we believed that BPD as a surgical therapy of T2DM in non-severely obese individuals should be indicated only with great caution. Furthermore, this investigation recommends that in evaluating the long-term outcomes following any bariatric/metabolic surgery in T2DM patients, not only the functional but mostly the clinical results should be taken into consideration.
